# Porphyrin and photodynamic therapy as tools against *Acinetobacter baumannii*

**DOI:** 10.1007/s10482-026-02413-8

**Published:** 2026-08-31

**Authors:** Isabela Dadda dos Reis, Marcelle Oliveira Garcia, Suelen Lopes Lussanrriaga, Kamila Furtado da Cunha, Bernardo Almeida Iglesias, Daniela Fernandes Ramos

**Affiliations:** 1 Laboratório de Desenvolvimento de Novos Fármacos, Faculdade de Medicina, Universidade Federal do Rio Grande (FURG), Rua General Canabarro, 102, Rio Grande, Rio Grande do Sul Brazil 96200-200; 2 Departamento de Química, Laboratório de Bioinorgânica e Materiais Porfirínicos, Universidade Federal de Santa Maria (UFSM), Avenida Roraima, 1000, Santa Maria, Rio Grande do Sul Brazil; 3 Departamento de Química, Grupo de Pesquisa em Fotobiologia e Macrocíclos Porfirínicos, Universidade Federal de Santa Catarina (UFSC), Florianópolis, Santa Catarina Brazil

**Keywords:** Reactive oxygen species (ROS), Antimicrobial resistance, Photoinactivation

## Abstract

Since the 1990s, *Acinetobacter baumannii* has become a major global concern owing to its high incidence in hospital-acquired infections. Although antimicrobial resistance is a natural process, it has been enhanced by the widespread use of antimicrobials in human and animal healthcare and is considered a global priority in the development of new drugs. Therefore, antibacterial photodynamic therapy has gained prominence for treating infections caused by multidrug-resistant microorganisms. This involves the use of photosensitizers, which, in the presence of light radiation at the appropriate wavelength, form reactive oxygen species, causing oxidative damage to bacterial cells. This review aims to critically evaluate the available literature on the efficacy of antimicrobial photodynamic therapy using porphyrins as photosensitizers against strains of *A. baumannii*. A search was conducted in five databases using the following keywords: “*Acinetobacter baumannii*,” “photoinactivation,” and “porphyrin.” Eight articles from different periods were selected, evaluating *in vitro* and *in vivo* assays at different concentrations and light incidence. Different concentrations of porphyrins interact differently with wavelengths, resulting in variable photosensitizer behavior, indicating that activity is also linked to virulence mechanisms, such as biofilm formation. Regarding multidrug-resistant strains, no relationship was observed between activity and resistance; however, higher concentrations of the compound were required in minimum inhibitory concentration assays. The compiled data provide important insights into the potential of porphyrins as alternatives to available antimicrobials and evaluate different parameters that influence their activity.

## Introduction

The first cases of infection by *Acinetobacter* spp. appeared between the 1960s and 1970s, when it was considered a low-virulence pathogen with little clinical significance (Shi et al. 2024). Beginning in the 1990s, the genus became a cause for concern when cases of nosocomial infections were associated with it; among them, the species *Acinetobacter baumannii* is responsible for approximately 80% of hospital-acquired infections. These Gram-negative aerobic coccobacilli belong to the *Moraxellaceae* family and can survive for long periods on dry surfaces, in the nosocomial environment, and on medical equipment, favoring hospital-acquired spread. Furthermore, they have a high capacity to develop resistance to antibiotics through intrinsic and acquired mechanisms, the main one being the production of beta-lactamases, enzymes that degrade their first-line therapeutic option, beta-lactam antimicrobial (Queiroz et al. 2022).

The emergence of resistance to antimicrobial agents is a natural process in bacteria that has been accelerated by the indiscriminate use of antibiotics in human and animal healthcare. This process has been causing increased morbidity and mortality, especially in underdeveloped countries. According to the World Health Organization (WHO), *A. baumannii* is classified as a critical priority pathogen in the global priority list for antibiotic research and development. This classification is primarily attributed to its high level of antimicrobial resistance, particularly strains resistant to carbapenems (carbapenem-resistant *A. baumannii*), states that, without radical innovation in antimicrobials, modern medicine as we know it will likely disappear (Shi et al. 2024; WHO. 2024).

This Gram-negative bacterium is a significant challenge in hospital settings, particularly in intensive care units, as it can develop resistance to multiple classes of antimicrobial drugs. This resistance can be due to various mechanisms, such as enzyme production (*β*-lactamases, such as AmpC, OXA-type, and metallo-*β*-lactamases) and efflux pumps (AdeABC, AdeIJK, and AdeFGH), which are responsible for the ineffectiveness of penicillins, cephalosporins, carbapenems, aminoglycosides, and fluoroquinolones, as well as their ability to form biofilms. Therefore, therapeutic options are becoming limited, reinforcing the urgent need for new treatment strategies, such as the development of new drugs (Shi et al. 2024; Serretiello et al. 2025).

Antibacterial photodynamic therapy has gained prominence in the search for alternatives to treat infections caused by multidrug-resistant (MDR) microorganisms. This approach relies on the use of a photosensitizer that, when exposed to light radiation of an appropriate wavelength, acquires antimicrobial photoactivity through the formation of reactive oxygen species (ROS), causing oxidative damage to membranes and cytotoxicity in bacterial cells (Vinagreiro et al. 2020; Carolina et al. 2025; Naskar and Kim 2023; Allamyradov et al. 2024).

**Fig. 1:**
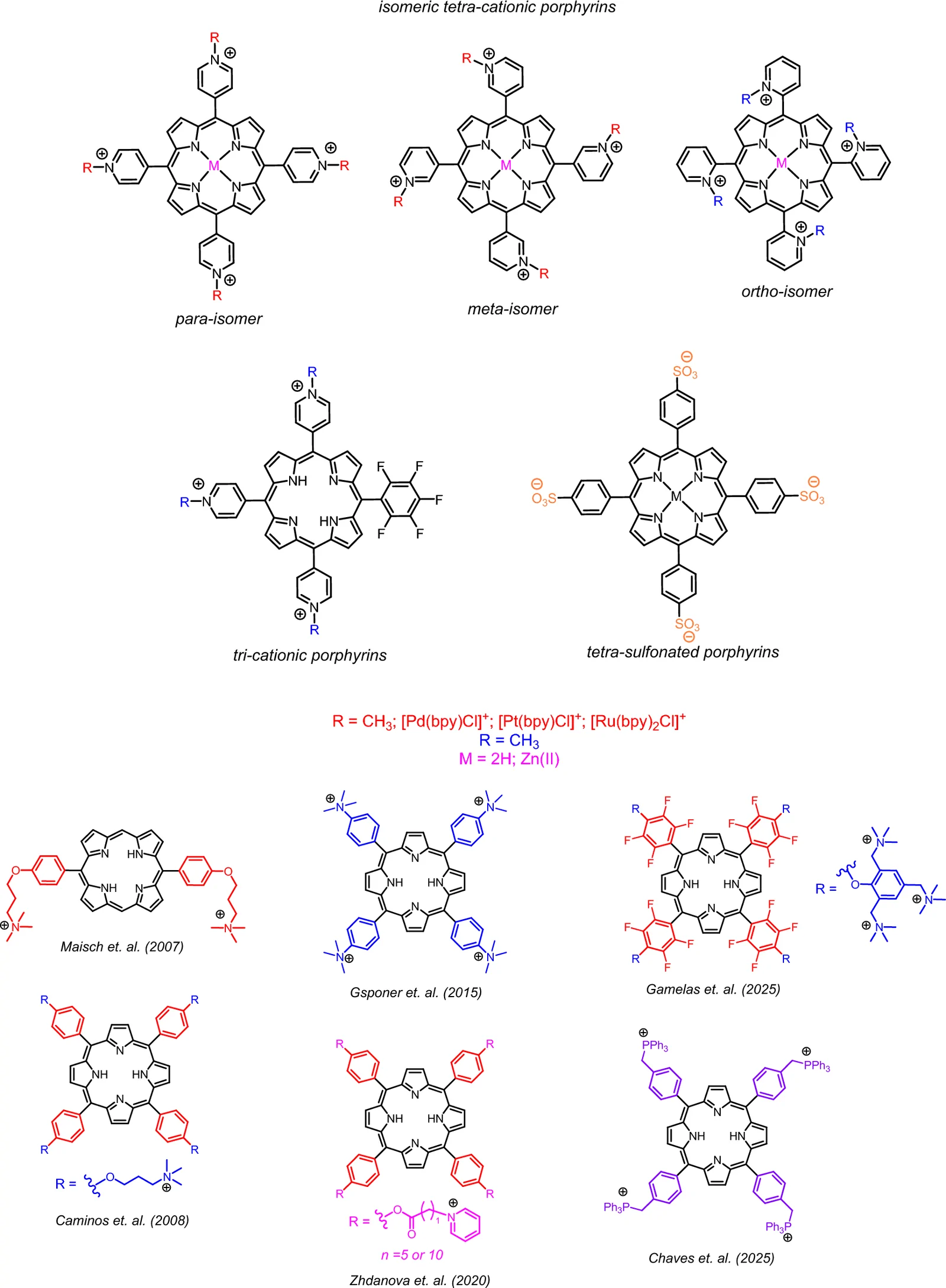
Some examples of porphyrin derivatives used in antimicrobial photoinactivation studies. Structural modification are highlighted in different colors represent chemical structure

Therefore, porphyrins are compounds with important photobiological activities because of their tetrapyrrolic structure, which provides adequate space for the insertion of a central metal ion. This allows the coordination of other ligands in the axial positions, enabling the compound to produce various ROS, such as singlet oxygen (O_2_), and certain radicals, such as hydroxyl (·OH) and superoxide (O_2_^·‒^) (Nitzan and Ashkenazi 2001; Oliveira et al. 2015). The search for porphyrin derivatives containing peripheral charges may be attractive for this application. In this regard, cationic or anionic porphyrins are of interest because they have high solubility in aqueous media, are usually photostable, and are good generators of ROS after light irradiation (Yuan et al. 2017; Caprara et al. 2022). Figure 1 highlights the structures of porphyrin compounds commonly used in antimicrobial photodynamic therapy (Zhdanova et al. 2020; Daniel 2008; Chaves et al. 2025; Gsponer et al. 2015; Gamelas et al. 2025; Maisch et al. 2007).

The efficacy of photodynamic therapy as an antibiotic alternative has already been well documented in the literature against several other bacteria, such as methicillin-resistant *Staphylococcus aureus* (MRSA) and methicillin-susceptible *Staphylococcus aureus* strains (Kasehf et al. 2013; Maisch et al. 2005; Grinholc et al.. 2008; Fekrazad et al.. 2014; Miyabe et al. 2011; Tang et al. 2007), *Escherichia coli* (Kashef et al. 2012; Grafe et al. 2004), *Pseudomonas aeruginosa* (Donnely and McCarron et al. 2007; Pourhajibagher et al. 2016), *Enterococcus faecalis* (Soukos et al. 1998; Eduardo et al. 2025), *Porphyromonas gingivalis* (Soukos et al. 1998; Love et al. 2007), *Aggregatibacter actinomycetemcomitans*, *Fusobacterium nucleatum*, *Prevotella intermedia*, and *Streptococcus sanguis* (Love et al. 2007). Additionally, photodynamic therapy has already shown success in reducing the minimum inhibitory concentration of antibiotics such as azithromycin, imipenem, ciprofloxacin, and gentamicin against *A*. *baumannii* (Kasehf and Yahyaei 2014). It can also alter the resistance profile of gentamicin-resistant MRSA strains, making them susceptible (Sperandio et al. 2014).

However, numerous existing studies demonstrating the efficacy of antimicrobial photodynamic therapy present variable or even hidden parameters of concentration, time, and type of irradiation, thereby generating conflicting information that hinders the establishment of clinical and scientific protocols (Nitzan et al. 1998). Therefore, the search for new antimicrobial technologies against *A*. *baumannii* is crucial, preferably mechanisms against which the bacterium is unable to develop resistance. In this scenario, photoinactivation appears to be a viable and accessible option for developing new protocols. Therefore, this review aims to evaluate the available literature on the efficacy of antimicrobial photodynamic therapy, using porphyrins as a photosensitizer, against *Acinetobacter baumannii* strains.

## Materials and methods

Five databases were searched: Scientific Electronic Library Online (Scielo), Coordination for the Improvement of Higher Education Foundation (CAPES), Virtual Health Library (BVS Brasil), ScienceDirect, and PubMed. The following keywords were used: “*Acinetobacter baumannii*,” “photoinactivation,” and “porphyrin.” Initially, 97 scientific articles published in the last 30 years were found.

**Scheme 1:**
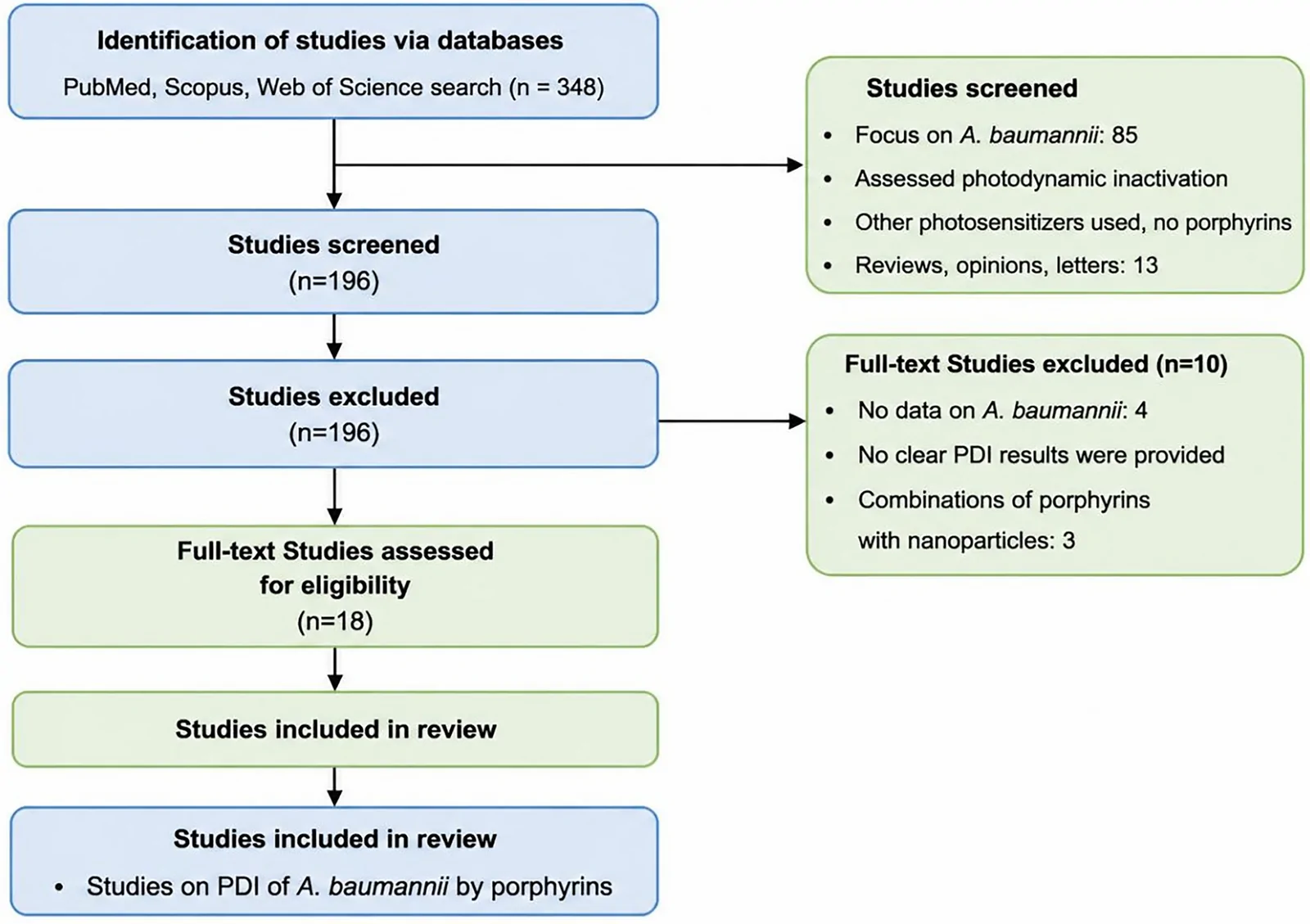
Selection process for research related to photodynamic inactivation of *Acintobacter baumannii* by some porphyrins

Studies including case reports, literature reviews, and those that did not use porphyrins as photosensitizing agents, did not use light exposure in the methodology, or did not perform tests against bacteria of the genus *Acinetobacter* were excluded. Only experimental *in vitro* and *in vivo* studies that performed tests including porphyrins as photosensitizing agents, light exposure in the methodology, and whose tests included bacteria of the genus *Acinetobacter* were considered. After applying the criteria, eight scientific articles were selected (Scheme 1).

## Results and discussion

Table 1 presents the main results evidenced by the scientific articles included in this work. The first studies involving the photoinactivation of *A*. *baumannii* were published at the end of the twentieth century. In 1998, Nitzan et al. (Nitzan et al. 2001) obtained positive results, generating important discussions regarding bacterial photoinactivation using a tungsten lamp (140 W/m^2^) for 90 min on Petri dishes containing culture medium inoculated with the bacteria. When testing different combinations of porphyrins with the polymyxin nanopeptide (PMNP) at 200 µmol/L, the authors found inhibitory effects of 60%, 88%, and 90% against *A. baumannii* with Mg-tetrabenzoporphyrin and Cd-texaphyrin (Cd-Tx) and deuteroporphyrin (Dp), respectively.

**Table 1:** Results of scientific articles included in review, in chronological order

Authors	Photosensitizer	*λ*_exc_	Irradiance (*E*)^╫^	Irradiation Time (min)	Bacteria Strain	Antimicrobial Potential
Nitzan et al. (Nitzan et al 1998)	Deuteroporphyrin (Dp), Mg-tetrabenzoporphyrin and Cd-texaphyrin (Cd-Tx) combined with nonapeptide polymyxin B	660 nm	140 W m^−2^	180	A. baumannii*	Inhibitory effect of 88% with Cd-Tx, 90% with Dp and 60% with Mg-tetrabenzoporphyrin
Ashkenazi et al. (Ashkenazi et al. 2003)	ACS-2-Butylhydroxy-toluene hematoporphyrin and ACS-3-propyl-gallate hematoporphyrin	407–420 nm	20 mW cm^−2^	Not included	A. baumannii*	Total eradication of the bacteria with ACS-2 at a concentration of 38.6 µM at 58 J/cm^2^, with a drop of 1.6 × 10^8^ bacteria/mL. With ACS-3, bactericidal action was obtained at 20 µM at 5 J/cm^2^ or with 1 µM at 40 J/cm^2^
Ashkenazi et al. (Ashkenazi et al. 2006)	Tetramethyl-pyridyl porphyrin	407–420 nm	20 mW cm^−2^	0.6	*A. calcoaceticus* BD 413	A survival percentage of 39% of *A*. *calcoaceticus* and 10% of *A. calcoaceticus *RecA( −) was obtained at 0.73 µmol/L. At 14.6 µmol/L, *A. calcoaceticus* RecA( −) exhibited 8% survival
Carpenter et al. (Carpenter et al. 2012)	Cellulose nanocrystals modified with porphyrin-derived photosensitizer	400–700 nm	65 mW cm^−2^	15–30	ATCC 19606; ATCC BAA-1605	A survival percentage of 6 logs was obtained after 15 min of exposure
Yuan et al. (Yuan et al. 2017)	Cationic amino acid porphyrin 4I (OS 4I)	650 nm	3 mW cm^−2^	30	A. baumannii*	MIC** of 3.9 µM and MBC*** of 7.8 µM for the MDR strain. MIC of 1.95 µM and MBC of 3.9 µM for the susceptible strain, presenting a survival fraction of 10^−4^ at 2 µM PS 4I, while the MDR strain had a survival fraction of 10^−4^ at 4 µM PS 4I. Group E (treated with photosensitizer and illumination) of rats had a survival rate of 80% after 22 days, much higher than the other groups, except for group A (uninfected), which had a rate of 100%. Tissue analysis showed that group E had a reduced number of bacteria until day 4 after infection (bacterial survival fraction of 10^−6^) and resumption of growth thereafter
Anane et al. ( Anane et al. 2020)	Protoporphyrin IX and methylene blue	625 nm	33 W cm^−2^	15	A. baumannii*	The concentration of 10 µM showed a reduction in cell viability of 3.5 log CFU/mL, while a concentration of 20 µM resulted in a reduction in cell viability of close to 6 log CFU/mL. A significant relationship was also observed in the destruction of preformed bacterial biofilms exposed to 1 µM porphyrin IX for 15 min (dose 30 J/cm^−2^)
Fayyaz et al. (Fayyaz et al. 2021)	Cationic porphine (TMPyP) and (4-N,N,N-trimethylanilinium) porphyrin (TAPP) implanted on cellulose surface	100 W tungsten lamp	0.36 mW cm^−2^	30	*A. baumannii*	TMPyP and Zn-TAPP at a concentration of 60 μg/mL and TAPP at a concentration of 15 μg/mL were 99.99% bactericidal
Caprara et al. (Caprara et al. 2022)	Meso-tetra(4-N-methyl-pyridyl)porphyrin (H_2_TMePyP^+^) and meso-tetra(4-sulfonato-phenyl)porphyrin (H_2_TPPS^−^)	400–800 nm	50 mW cm^−2^	90	A. baumannii*	Not antibacterial activity was identified, without illumination, for any of the two porphyrins evaluated, at 19.74 µM and 23.35 µM. Using H_2_TMePyP^+^, MICs of 4.93 and 0.61 μM were obtained with illumination against ciprofloxacin-resistant and amikacin-sensitive strains (Strain 1 and 6, respectively). With H_2_TPPS^−^, MIC of 23.35 μM was obtained with illumination against the same strain, respectively. Furthermore, a reduction of up to 35.6% was observed in biofilm formation at a concentration of 1.22 mM
Vinagreiro et al. (Vinagreiro et al. 2020)	5,10,15,20-(1,3-dimethylimidazol-2-yl) porphyrinate zinc (II) tetraiodide (IP-4-Zn+)	400-700 nm	30 mW/cm^2^	Not included	A. baumannii*	The IP-4-Zn^+^- stood out for promoting a reduction greater than 6 log_10_ CFU/mL in *A. baumannii* using a concentration of 5 nM, under irradiation with white light (400–700 nm) and a light dose of 5 J/cm^2^
Domingos ( Domingos et al. 2025)	5,10,15,20- tetrakis (1,3-dimethylimidazol-2-yl) porphyrinate tetraiodide	400 nm	13,1 mW/cm^2^	30	A. baumannii*	Biodegradable poly (lactic acid) (PLA) films with 5,10,15 - Tris ((4-Trifluormethyl)phenyl)-20-(1,3-dimethyl-1*H-*imidazol-2-yl) porphyrin iodide (PLA-3) were developed. Films containing 0.20% (w/w) photosensitizer (+ PLA-3) showed activity against *A. baumannii* after irradiation with a blue LED (415 nm) and a light dose of 23.5 J/cm^2^, showing a reduction of approximately 7log CFU/mL

*clinical isolates; **minimum inhibitory concentration; ***minimum bactericidal concentration; ^**╫**^calculated according to Diffey (2002)

**Fig. 2:**
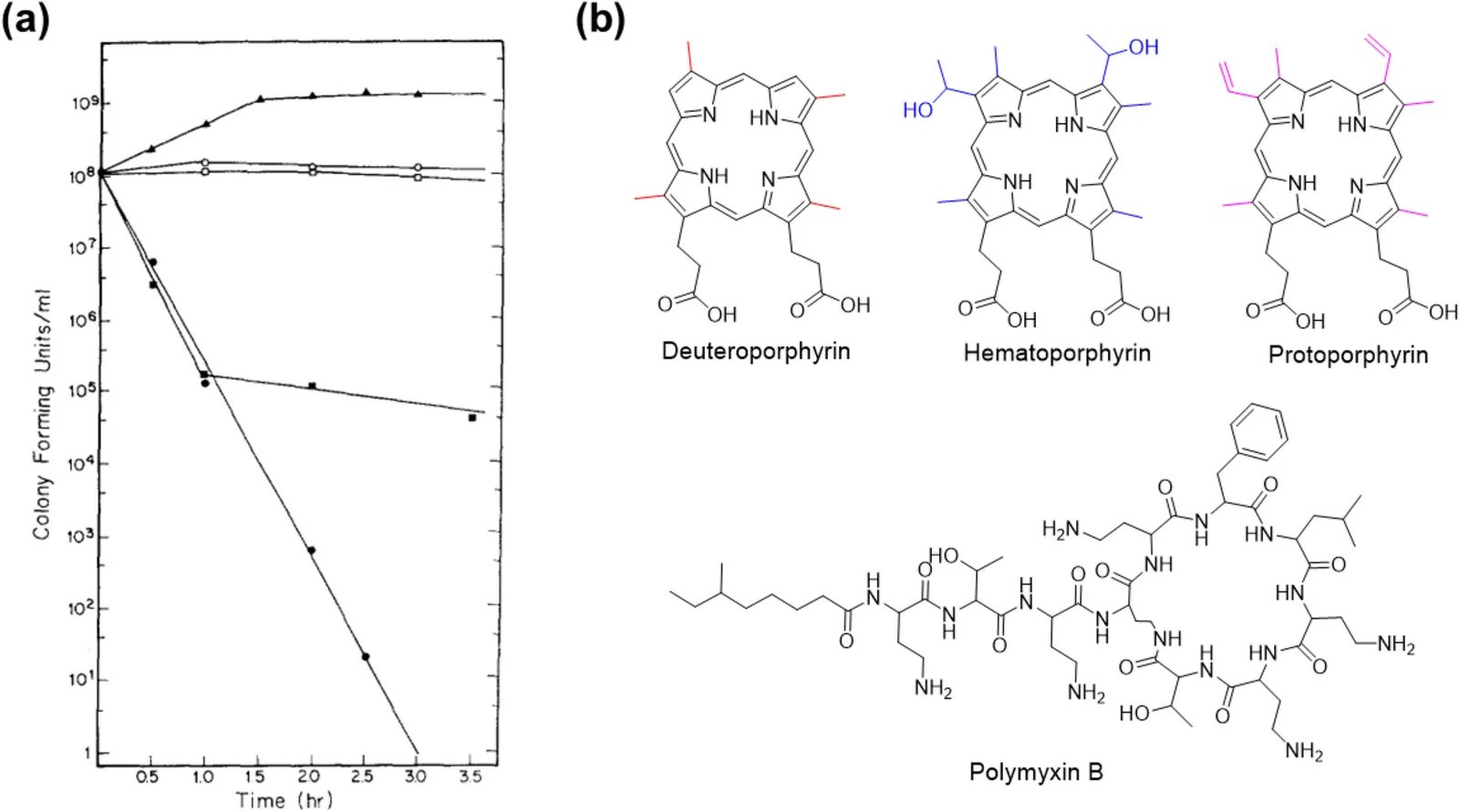
**(a)** Viability of *Acinetobacter baumannii* treated by TMPyP or deuteroporphyrin (Dp) in the presence of polymyxin B sulfate (PMNP) in serum (15 mg/ml protein) and (**b)** structural representation of porphyrins and PMNP molecules used in this study. Reproduced with permission from reference (Ashkenazi et al. 2003)

In the same study, the authors highlighted the influence of the culture medium used to evaluate antibacterial activity. Bacterial photoinactivation tests were performed in different media, one containing 15 mg/mL fetal bovine serum and the other containing 15 mg/mL bovine serum albumin. In the medium containing fetal bovine serum, the combination of Dp and PMNP (34 µmol/L) induced bacterial death at seven orders of magnitude higher than cationic porphine (TMPyP) at 32 µmol/L alone. In contrast to these findings, in the medium containing serum albumin, TMPyP promoted bacterial death at seven orders of magnitude higher than the combination of Dp and PMNP. These results demonstrate that the type of protein present in the medium affects the photoinactivating properties of the compounds (Fig. 2).

The same research group, evaluating the hydrophilic cationic porphyrin 5,10,15,20-tetra(4-N-methylpyridyl) (TMPyP), including in the absence of the membrane-permeable peptide, polymyxin nanopeptide (PMNP), against MDR *A*. *baumannii* isolates, demonstrated bacterial eradication under the following conditions: a concentration of 29.4 µmol/L of TMPyP and 2 J/cm^2^ of blue light (400–450 nm), 15 J/cm^2^ of green light (480–550 nm), and 32 J/cm^2^ of red light (600–700 nm). Even with a reduced concentration of TMPyP (3.7 µmol/L), the bactericidal effect could be guaranteed with 9 J/cm^2^ of blue light (wavelength of 407 nm) (Nitzan and Ashkenazi 2001). Furthermore, the authors observed the results with exposure to different types of light. They concluded that to obtain bactericidal action on bacteria with the same concentration of TMPyP, an energy eight times greater than that of green light and 16–20 times greater than that of red light is required compared with blue light. Therefore, this study corroborates other studies that argue that blue light (400–475 nm) is more efficient in photoinactivation (Nitzan and Ashkenazi 2001; Ashkenazi et al. 2003, 2006). This is because porphyrins exhibit maximum light absorption at these wavelengths, although red light illumination would be ideal for clinical treatment, as it penetrates tissues better (Nitzan and Ashkenazi 2001).

Among the studies analyzed, those that used blue illumination (407–420 nm) obtained better results than those that used other wavelengths. In addition to the results already presented by Nitzan et al. (Nitzan and Ashkenazi 2001), Ashkenazi et al. 2006 achieved 98% bacterial kill using low concentrations of photoinactivator (14.6 µmol/L) and low blue light fluence (4 J/cm^2^). However, Yuan et al. (Yuan et al. 2017) achieved bacterial inactivation of only 3.77 log CFU/mL using red light (650 nm) at a fluence (6 J/cm^2^) and concentration (3.9 µM) of photoinactivator. In contrast, Anane et al. (Anane et al. 2020) observed greater bacterial inactivation (6 log) using white light (560–780 nm), but with a higher irradiance (fluence of 30 J/cm^2^) and a higher porphyrin concentration (20 µM).

**Fig. 3:**
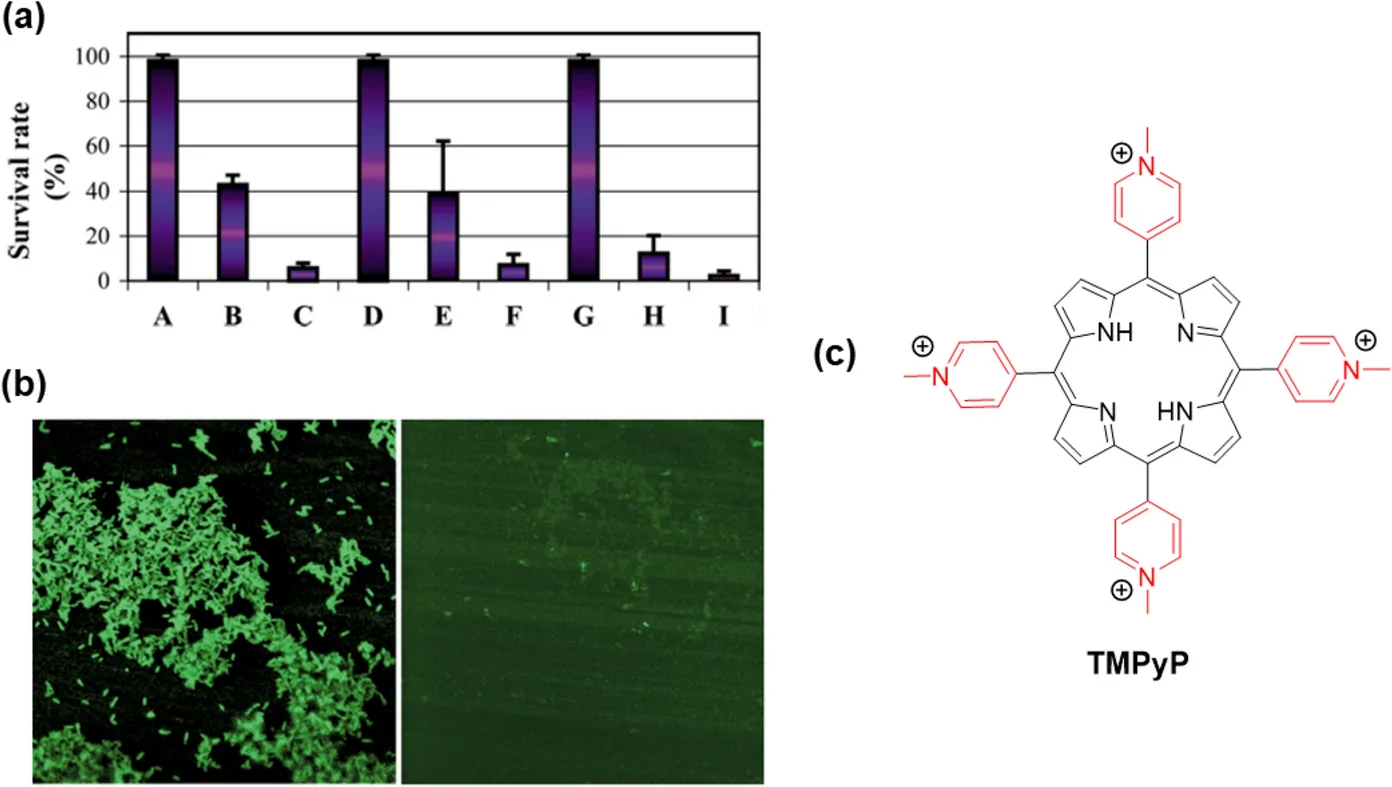
**(a)** Viability of *E. coli B, A. calcoaceticus* BD 413, and the *A. calcoaceticus* RecA (‒) mutant after photo-treatment. Bacterial cultures were treated with 0.73 μM TMPyP (columns B, E, and H, respectively) or with 14.6 μM TMPyP (columns C, F, and I, respectively). All cultures were illuminated with 4.0 J/cm^2^ blue light at 407 to 420 nm. Illuminated cultures of each bacterium with no photosensitizer served as controls (columns A, D, and G, respectively); (**b)** Confocal fluorescence microscopy of photosensitized *E. coli B*. Cultures were treated with 0.73 μM TMPyP and illuminated with 4 J/cm^2^ at 407 to 420 nm blue light. Cultures were treated with mouse anti-*E. coli* RecA primary antibodies. FITC-conjugated goat antimouse secondary antibodies were reacted with the cell (left panel). Photosensitized cells that were reacted with only the secondary antibody were used as control (right panel) and **c** structural representation of porphyrin TMPyP used in this study. Reproduced with permission from reference (Ashkenazi et al. 2006)

Ashkenazi et al. (Ashkenazi et al. 2003) concluded that phototreatment with blue light (407–420 nm) for a short period was sufficient to eradicate bacterial cells using TMPyP, a hydrophilic cationic photosensitizer. When exposing cultures of wild-type *A*. *calcoaceticus* and *A*. *calcoaceticus* RecA( −)-mutant to a light intensity of 4 J/cm^2^ after treatment with 0.73 µmol/L TMPyP, the viability was reduced by 61% and 90% for each bacterial isolate, respectively. With treatment using 14.6 µmol/L TMPyP at a light intensity of 4 J/cm^2^, the reduction in viability was even more significant, reaching 92% and 98%, respectively (Fig. 3).

Some studies have reported that *A. baumannii* produces endogenous porphyrins as intermediates in the production of heme groups, which are essential for processes such as cellular respiration. Recently, it has been proposed that exposure to 402–440 nm (blue light) to promote the reduction of bacterial populations by photodynamic therapy, assessed at 35 mW/cm^2^ irradiance, leads to ROS generation and causes cellular damage (Ashkenazi et al. 2006)

**Fig. 4:**
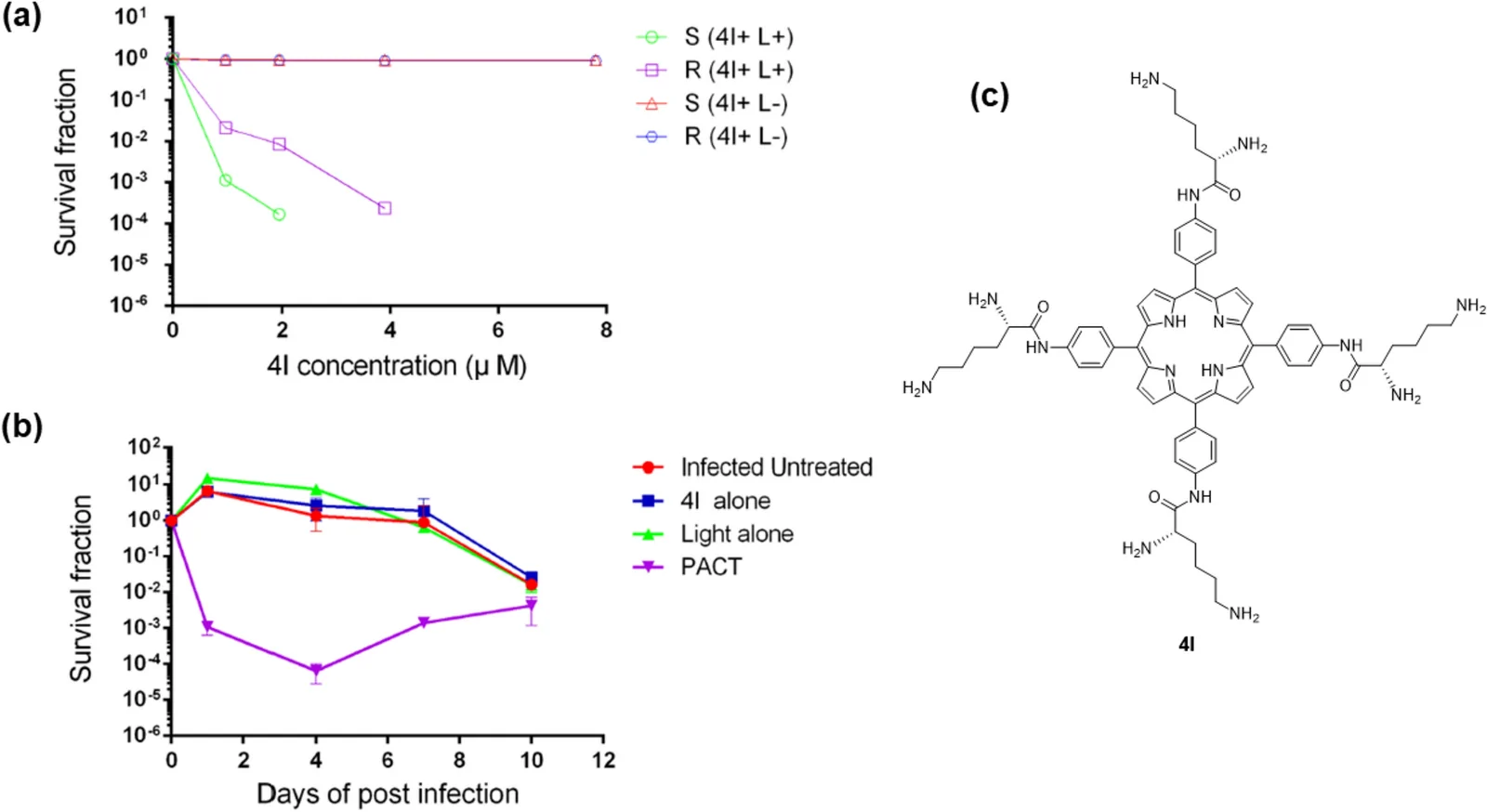
**(a)** Survival fraction of two strains treated by different concentrations of 4I with or without illumination (6.0 J/cm^2^ for 30 min). S, sensitive strain; R, multidrug strain; 4I+ L+, treated by 4I combined with light; 4I+ L−, treated by 4I not combined with light; (**b)** Survival fraction of *A. baumannii* isolated from excised tissues of mice in four different treatment groups at determined time points and **c** structural representation of porphyrin 4I used in this study. Reproduced with permission from reference (Yuan et al. 2017)

When obtaining positive results against MDR *A*. *baumannii*, Yuan et al. (Yuan et al. 2017) evaluated a cationic porphyrin conjugated with four lysine groups, 5,10,15,20-tetrakis(4-((s)− 2,6-diaminohexanamido)-phenyl) porphyrin, combined with red light irradiations (650 nm) at 6 J/cm^2^, against *A*. *baumannii* (isolated) *in vitro* and* in vivo *assays. Initially, the authors observed bacterial inactivation of 1.79, 2.15, and 3.77 log CFU/mL with concentrations of 0.975 µM, 1.95 µM, and 3.9 µM, respectively. The Minimun Inhibitory Concentration (MIC) and Minimum Bactericidal Concentration (MBC) found were 3.9 µM and 7.8 µM for the sensitive and MDR strains, respectively, suggesting that bacterial resistance has some relevance, even if not yet elucidated, in the efficacy of photoinactivation. *In* *vivo* assays, the effect of this porphyrin was evaluated in mice with deep wounds infected with *A*. *baumannii*. For this purpose, the infected wounds of the animals were excised and treated with 40 µM and a fluence of 50 J/cm^2^. After treatment with photoinactivation and 5,10,15,20-tetrakis(4-((s)− 2,6-diaminohexanamido)-phenyl) porphyrin, a 2.89 log reduction in bacterial count was observed. In contrast, the group treated only with the compound or only with irradiation exhibited an increase in bacterial count. Furthermore, the groups treated with photodynamic antimicrobial chemotherapy demonstrated faster wound resolution than the control groups, promoting wound healing approximately 5 days earlier than the group treated only with illumination without the association of photosensitizing compounds and 10 days earlier than the other groups (Fig. 4).

**Fig. 5:**
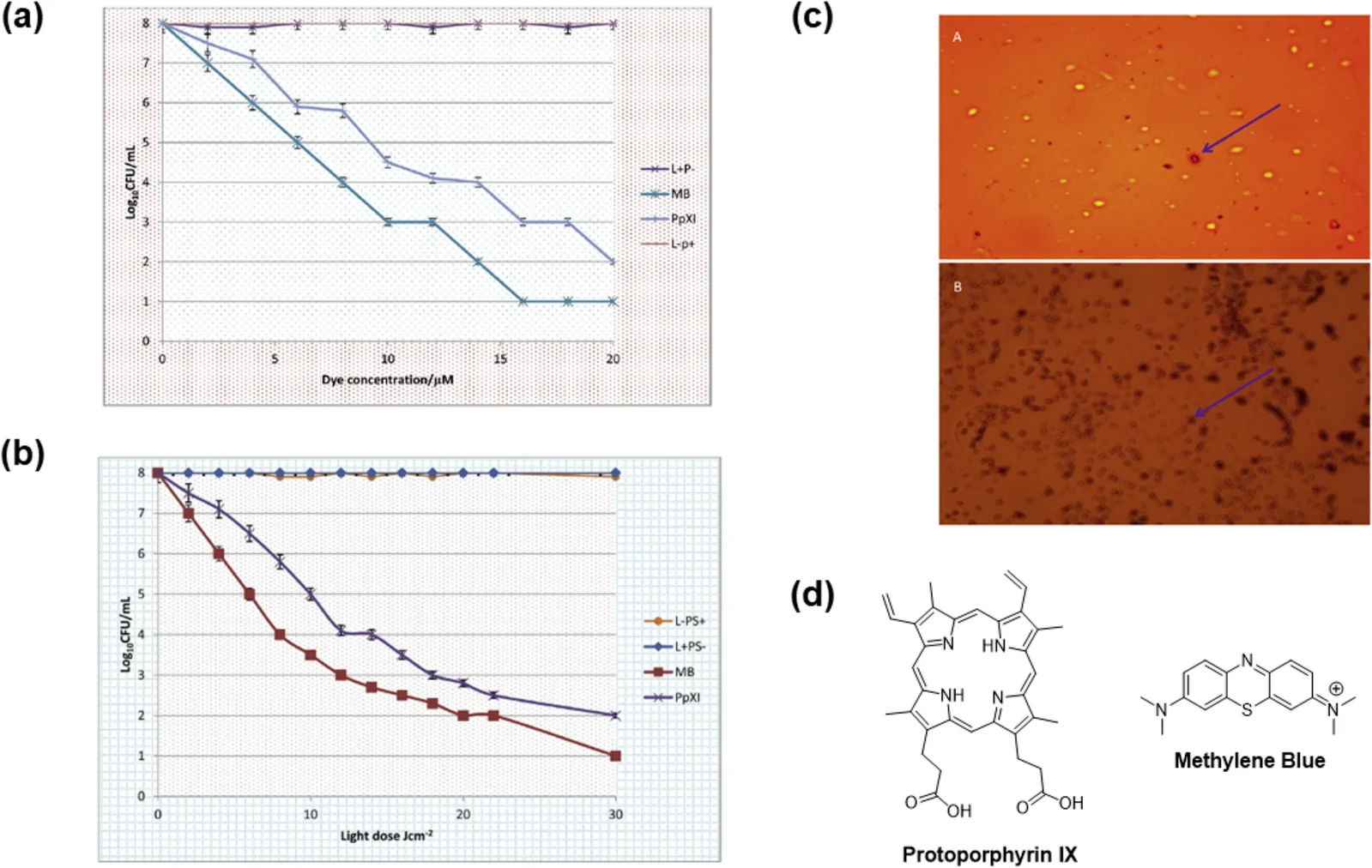
**(a)** Antimicrobial photodynamic inactivation of different Protoporphyrin IX and Methylene blue concentrations ranging from 1.0 μM to 20 μM on the viability of *A. baumannii* ATCC 19606. Dark incubation time 15 min, and the light dose 30 J/cm^2^; (**b)** Energy–dose response for *in vitro* photodynamic inactivation of *A. baumannii* ATCC 19606 with Methylene blue (squares) and Protoporphyrin IX (cross marks). The photosensitizers dose was 10 μM, and the irradiation wavelength was 652 nm; (**c)** Localization of Methylene blue and Protoporphyrin IX in *A. baumannii* ATCC 19606 biofilm cells. *A. baumannii* ATCC 19606 biofilms were incubated for 15 min with Methylene blue. The distribution of Methylene blue was examined by fluorescence microscopy. Arrow showing the accumulation of photosensitizer, where A=Methylene blue and B=Protoporphyrin IX and (**d)** structural representation of dyes used in this study. Reproduced with permission from reference (Anane et al. 2020)

More recently, another study conducted by Anane et al. (Anane et al. 2020) evaluated the activity of the tungsten lamp (560–780 nm) by testing the effect of 3,7,12,17-tetramethyl-8,13-divinyl-2,18-porphinedipropionic acid (protoporphyrin IX) in mediating photodynamic therapy against MDR strains of *A*. *baumannii*. The authors achieved bacterial reductions of 5 log_10_ when protoporphyrin IX was combined with illumination. However, in the absence of porphyrin, no significant reduction was observed in bacterial cells in the non-irradiated and irradiated groups. Similarly, the concentration of protoporphyrin IX appears to be crucial for the final outcome, as 10 µM exhibited a reduction in cell viability of 3.5 log CFU/mL, while 20 µM resulted in a reduction in cell viability close to 6 log CFU/mL. Furthermore, Anane et al. (Anane  et al. 2020) demonstrated a significant relationship (p‹0.05) in the destruction of preformed bacterial biofilms exposed to 1.0 μM protoporphyrin IX for 15 min (dose: 30 J/cm^2^) (Fig. 5). Another study conducted with water-soluble proteins demonstrated that the eradication capacity of preformed bacterial biofilms after 24 h was dependent on porphyrin concentration, as 2.44 μM meso-tetra(4-N-methyl-pyridyl)porphyrin (H_2_TMePyP^+^) could promote biofilm destruction (destruction of 8% of the biofilm).

Caprara et al. (Caprara et al. 2022) believe that photoinactivation therapy associated with porphyrin alone is effective only in the first stages of biofilm formation, because in their study, cationic porphyrin inhibited 35.3% of the biofilm formation of *A*. *baumannii* ATCC 19606. They also observed a 20% increase in the inhibitory potential of biofilm formation in this strain when associated with LED illumination with white light (400–800 nm) at 270 J/cm^2^ for 90 min.

In the context of different light exposures, Ashkenazi et al. (Ashkenazi et al. 2003) investigated the use of 3,7-bis(2-carboxyethyl)− 2,8,12,17-tetramethyl-13,18-bis[1-(3,5-di-tert-butyl-1-4-hydroxy-benzyloxy)ethyl]porphyrin (ACS-2) and 3,7-bis(2-carboxyethyl)− 2,8,12,17-tetramethyl-13,18-bis[5-propoxycarbonyl-3-4 dihydroxyphenyloxy)ethyl]porphyrin (ACS-3) combined with blue light exposure (401–410 nm) against MDR *A*. *baumannii* isolates. The photosensitizer ACS-2 demonstrated photoinactivation capacity only when associated with polymyxin B (500 µM). Bacterial eradication was observed in the presence of 38.6 µM ACS-2 and a light fluence of approximately 58 J/cm^2^. In contrast, the photosensitizer ACS-3 exhibited significant photobactericidal activity without the need for adjuvant antibiotics, promoting reductions of up to seven orders of magnitude in the viability of *A*. *baumannii*. This effect was obtained with 20 µM ACS-3 and 5 J/cm^2^, or with 1 µM ACS-3 and a light fluence of 50 J/cm^2^.

**Fig. 6:**
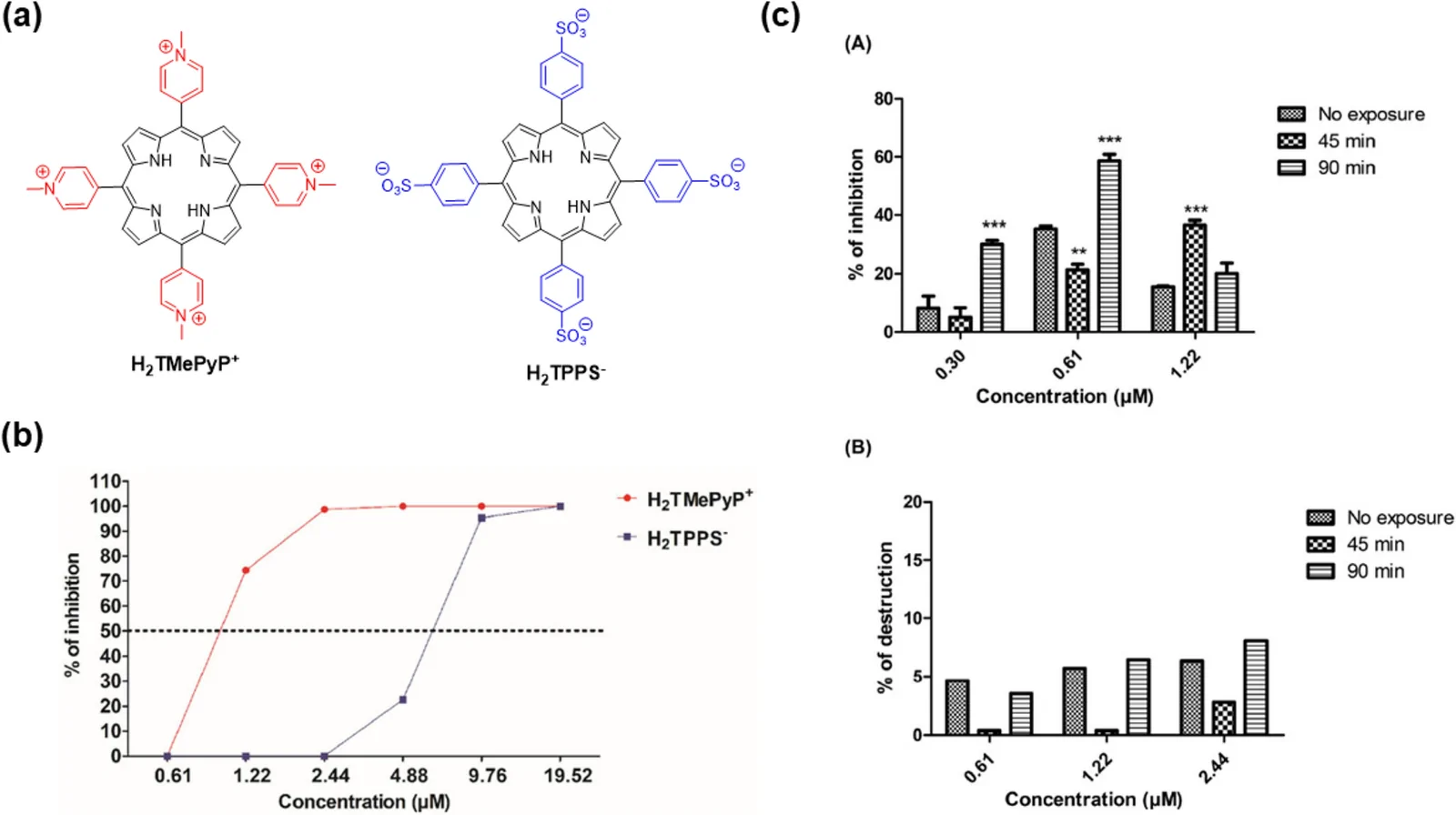
**(a)** Structural representation of cationic and anionic porphyrins used in this study; (**b)** Antimicrobial photoactivity curve of two porphyrins against *Acinetobacter baumannii* (ATCC 19606) expressed in percentage of inhibition regarding bacterial control without treatment at a fluence rate of 50mW/cm^2^ and total light dosage of 270 J/cm^2^ for 90 min and (**c)** Representation of the capacity of the H_2_TMePy^+^ in (A) biofilm inhibition and (B) destruction in *A. baumannii* strain ATCC 19606 for up to 90 min of white-light exposure. Reproduced with permission from reference (Caprara et al. 2022)

Caprara et al. (Caprara et al. 2022) successfully evaluated the water-soluble porphyrins *meso*-tetra(4-N-methyl-pyridyl)porphyrin (H_2_TMePyP^+^), cationic, and *meso*-tetra(4-sulfonato-phenyl)porphyrin (H_2_TPPS^−^), anionic, using white LED illumination (400–800 nm) for 90 min (270 J/cm^2^) against six *A*. *baumannii* isolates with different sensitivity profiles to the main drugs used in therapy. Under these conditions, the MIC values obtained with H_2_TMePyP^+^ ranged from 0.61 µM to 19.74 µM; with H_2_TPPS^−^, the MIC range was 11.67 µM to 23.35 µM; the concentrations capable of inhibiting 90% of the bacterial population (IC90) were 1.66 µM and 9.50 µM for H_2_TMePyP^+^ and H_2_TPPS^−^, respectively (Fig. 6) (Caprara et al. 2022).

Therefore, the combination of irradiation and photosensitizing compounds, such as porphyrins, is markedly more effective for bacterial eradication than light alone. The mechanism of photodynamic therapy relies on the intracellular accumulation of the photosensitizer and its ability to absorb light energy. Upon irradiation, photosensitizer molecules transition from the ground state to an excited state, enabling interactions with bacterial biomolecules and leading to ROS generation (Nitzan and Ashkenazi 2001; Fayyaz et al. 2020). In this context, the photosensitizer acts as a key mediator of the process, as its physicochemical properties and absorption spectrum can be precisely controlled, allowing optimization of the excitation wavelength according to the target tissue. Among these compounds, porphyrins stand out because of their well-established photochemical and photobiological properties, which make them highly suitable for photodynamic applications (Yuan et al. 2017).

Although Ashkenazi et al. (Ashkenazi et al. 2006) identified damage to the plasmid and chromosomal DNA of *A. baumannii* after treatment with 15 J/cm^2^, as well as DNA fragmentation after exposure to 30 J/cm^2^, other studies involving energy-dispersive X-ray spectroscopy revealed electrolyte disturbances in cells treated with photosensitizers and illumination, specifically in potassium, sodium, chloride, and magnesium ions, indicating that the damage obtained was related to the bacterial cell membrane (Ashkenazi et al. 2003, 2006). This evidence was reinforced when Ashkenazi et al. (Ashkenazi et al. 2006) found no significant damage to bacterial DNA through gel electrophoresis analysis, strengthening the hypothesis that cell death occurs because of impairment of Na^+^/K^+^ pumps, the cell membrane, and cytoplasmic enzymes due to ROS, as these structures lack repair mechanisms, unlike DNA, and strongly affect cell viability.

Fluorescence microscopy analyses by Anane et al. (Anane et al. 2020) demonstrated that photosensitizers accumulate predominantly within the bacterial cell and cell wall, without association with genetic material, suggesting a low mutagenic potential of porphyrins. Complementarily, Caprara et al. (Caprara et al. 2022) evaluated the role of ROS in modulating the antimicrobial activity of H_2_TMePyP^+^, highlighting that radicals, such as hydroxyl (·OH), superoxide (O_2_^·‒^), and hydroperoxyl (·OOH), maintained or reduced MIC values by up to fourfold. In contrast, the presence of non-enzymatic antioxidants, including ascorbic acid and ethylenediaminetetraacetic acid, significantly increased MIC values, likely owing to their protective effect on bacterial cell membranes (Caprara et al. 2022).

Antibacterial photodynamic therapy has been investigated for the treatment of various diseases for over five decades, with important results against MDR pathogenic bacteria, such as *Staphylococcus* spp., *Streptococcus* spp., *E. coli*, *P.s aeruginosa*, and, more recently, as demonstrated in this review, *Acinetobacter* spp. Its activity is based on the photosensitization of bacteria by exogenous compounds, such as porphyrins, leading to bactericidal effects driven by lethal oxidative stress upon irradiation with light at resonant wavelengths, typically within the visible spectrum (400–700 nm). These effects arise from type I and type II photochemical reactions, involving electron transfer from the excited triplet state to surrounding substrates or direct interaction with molecular oxygen (O_2_), resulting in the generation of reactive species, particularly singlet oxygen (^1^O_2_) (Angelino et al. 2017). Although several *in vitro* studies have corroborated the efficacy of photodynamic therapy in a wide variety of bacteria, more in-depth studies are needed to provide evidence regarding the intracellular pharmacokinetics and pharmacodynamics of photosensitizers, which would stimulate translational advances for the clinical setting.

Advancement in the development stages of new therapeutic strategies is needed to combat critical pathogens, such as *A*. *baumannii*; however, studies evaluating the potential of porphyrins against these bacteria are still in the early stages of this process. Therefore, we also searched for the use of porphyrins in photodynamic therapy in clinical trials against other microorganisms using the database (clinicaltrials.gov) and the keywords “porphyrins” and “infection.” To understand the challenges that may be faced in future stages of implementing porphyrins in photodynamic therapy to combat infections caused by *A*. *baumannii*, we selected trials that evaluated the activity of various porphyrins against different microorganisms.

The clinical potential of porphyrins has been demonstrated in several studies. For instance, Angelino et al. (Angelino et al. 2017) (NCT03379337) evaluated the fluorescence of porphyrins produced by oral microorganisms as a non-invasive biomarker for detecting dental plaques. The study showed that red fluorescence emission, detected using a portable blue light device (405 nm), was strongly correlated with biofilm accumulation, supporting the use of porphyrin fluorescence as a reliable tool for oral health monitoring.

Another significant advance is represented by the porphyrin derivative exeporfinium chloride (XF-73), a synthetic antimicrobial agent formulated as a nasal gel to reduce *Staphylococcus aureus* colonization in surgical patients. In a randomized, double-blind, placebo-controlled phase II clinical trial (NCT03915470) (Savelyeva et al. 2023), XF-73 demonstrated a substantial reduction in nasal bacterial load without associated adverse effects. Patients received four applications of XF-73 0.2% gel or placebo within 24 h before surgery, followed by an additional dose after wound closure. The primary endpoint, change in *S. aureus* load (log_10_ CFU/mL), revealed a mean reduction of − 2.84 log_10_ CFU/mL in the XF-73 group compared with −0.47 log_10_ CFU/mL in the placebo group, corresponding to an adjusted difference of approximately − 2.1 log_10_ CFU/mL. Notably, 83.7% of treated patients achieved a reduction of  ≥ 2 log_10_ CFU/mL or complete decolonization, compared with 25% in the placebo group. Additionally, the treatment exhibited an excellent safety profile, with no serious adverse events reported. These findings reinforce the potential of chemically modified porphyrins as fast-acting and safe antimicrobial agents, capable of overcoming conventional bacterial resistance mechanisms.

Furthermore, some current studies have been highlighting results of the antibacterial activity of chlorins, which are tetrapyrrolic pigments, partially hydrogenated porphyrins. Among then Choong-Won (Seo et al. 2022) demonstrated that after photodynamic therapy with Radachlorin, there were significant morphological changes in biofilms of multidrug-resistant *A. baumanii* isolates when exposed to a concentration of 1 mg/mL of Radachlorin for 1 h on 630-nm LED. In *Galleria mellonella* as biological model, Figueredo-Goi et al. (Figueiredo-Godoi et al. 2022) report the activity of a photosensitizer derived from chlorine e−6 (Fotoenticine—FTC) in comparing its effects with methylene blue (MB), in *A. baumannii* biofilms and burn infections in *G. mellonella.* As results were observed reductions ranging from 1.4 to 2 log_10_ CFU for FTC and from 2 log_10_ CFU for total inhibition for MB and *in vivo* model, only MB-mediated Antimicrobial Photodynamic Therapy (aPDT) had antimicrobial activity in burns, increasing larval survival by 35%. Dai (Dai et al. 2009) demonstrated that antimicrobial photodynamic therapy using the photosensitizer chlorine(e6)-polyethyleneimine (PEI-ce6) and red light significantly reduced bacterial viability in balb/c model, leading to a reduction of 3 log CFU when applied after infection and approximately 1.7 log CFU when initiated two days later, showing greater efficacy in the early stages of infection. These studies also demonstrate promising results on porphyrin derivatives, representing a potential target to be explored and applied against *A. baumannii*, as demonstrated *in vitro* and *in vivo*.

Owing to its potential use during the COVID-19 pandemic, the investigation of a synthetic porphyrin activated by sunlight as an antiviral strategy was proposed. Upon exposure to light, the molecule generates ROS capable of inactivating SARS-CoV-2 and modulating infection. Although innovative, this approach remains experimental, with no published clinical results to date, highlighting the challenge in demonstrating the potent photodynamic reactivity of porphyrins in safe and effective therapies in humans (NCT04371822) (Mendes 2008).

Supporting the findings discussed above, Savelyeva et al. (Savelyeva et al. 2023) demonstrated the potential of cationic porphyrins as antimicrobial and antiviral agents in photodynamic therapy, highlighting their broad-spectrum efficacy and versatility. These compounds exhibited strong *in vitro* photoinactivation against Gram-positive and Gram-negative bacteria, in addition to notable antiviral activity. The incorporation of multiple positive charges into the porphyrin structure significantly enhanced their affinity for negatively charged microbial membranes, thereby increasing photodynamic efficiency. Moreover, cationic porphyrins showed a pronounced ability to reduce biofilm-associated microbial viability, including in highly resistant species, such as *P. aeruginosa*. *In vivo* studies further confirmed their therapeutic potential, with certain derivatives effectively reducing bacterial load in *E. coli*-infected wounds, even at low concentrations and under moderate light doses.

The photodynamic antiviral activity of these molecules has been proven effective in inactivating model viruses, such as bacteriophage MS2, through irreversible structural and genomic damage induced by ROS generated during irradiation. This study highlights that the mechanism of action of porphyrins is nonspecific, which reduces the likelihood of developing microbial resistance, representing one of the greatest advantages of photodynamic therapy over conventional antimicrobials. Finally, the authors emphasize that photodynamic efficiency depends on several structural factors, such as the number of cationic groups, the presence of central metals (such as Zn^2+^ ions), the hydrophilic-hydrophobic balance, and the use of delivery systems, such as micelles, which favor cellular penetration and porphyrin stability (Mendes 2008).

When stimulated with the appropriate wavelength, the molecules of the photosensitizing compound transition to an excited state. From this, the molecules react with bacterial cells, forming radical ions that, through the transfer of electrons to oxygen, generate highly oxidizing ROS. ROS interact with sites of high electron density, such as DNA and cell membranes, initiating chain reactions that lead to damage at these sites and are cytotoxic (Mendes 2008).

Furthermore, the photoinactivation of Gram-negative bacteria remains more challenging than that of Gram-positive organisms, primarily because of the presence of an outer membrane that restricts the uptake of anionic compounds. In this context, cationic porphyrins exhibit superior efficacy, as their positive charge facilitates their interaction with and penetration through the negatively charged outer membrane and peptidoglycan layer (Caprara et al. 2022; Ashkenazi et al. 2003). These findings highlight the therapeutic potential of cationic porphyrins as promising alternatives for controlling microbial and viral infections, particularly in the context of increasing resistance to conventional antimicrobials (Mendes 2008).

## Conclusions

Research available in the literature on this issue presents data dating back to the last century, addressing various factors that influence the eradication process of *A*. *baumannii*. Despite limitations related to the limited number of studies and the lack of standardized protocols, current evidence consistently indicates that cationic porphyrins activated by blue light (400–420 nm) represent the most effective strategy for photoinactivation of *A. baumannii*, owing to their ability to maximize ROS generation. Treatment efficacy is further influenced by the light dose and irradiation time, whereas environmental factors—particularly protein content and medium composition—emerge as critical modulators of therapeutic performance, underscoring the need for protocol standardization to enable clinical translation. The findings indicate that blue light has greater bactericidal potential at lower radiation levels. Although data related to bacterial death and other virulence mechanisms are lacking, photosensitizing agents can eradicate biofilms in their early stages and do not exhibit mutagenic activity. In vivo assays revealed that their activity remained promising, with the ability to reduce the survival of *A*. *baumannii* in the wound and shorten tissue healing time.

The data presented suggest that porphyrins effectively combat MDR bacteria. However, resistance to this treatment, although not yet fully understood, requires the use of higher concentrations. Overall, porphyrins with a positive charge (cationic derivatives) demonstrate therapeutic potential in response to the growing global need for alternatives to available antibiotics. Some gaps still need to be filled to enhance the photodynamic activity of these photosensitizers, mainly regarding the appropriate chemical structure for this type of application. New studies and molecules are emerging in the current literature, with various research groups aiming to enhance the efficacy of photodynamic therapy at low irradiation dosages and prolong ROS actions generated during the photodynamic process.

## Data Availability

No datasets were generated or analysed during the current study.
